# Modeling Interactions among Individual P2 Receptors to Explain Complex Response Patterns over a Wide Range of ATP Concentrations

**DOI:** 10.3389/fphys.2016.00294

**Published:** 2016-07-13

**Authors:** Shu Xing, Matthew W. Grol, Peter H. Grutter, S. Jeffrey Dixon, Svetlana V. Komarova

**Affiliations:** ^1^Department of Physics, McGill UniversityMontreal, QC, Canada; ^2^Shriners Hospital for Children-CanadaMontreal, QC, Canada; ^3^Department of Anatomy and Cell Biology, Schulich School of Medicine and Dentistry, University of Western OntarioLondon, ON, Canada; ^4^Department of Physiology and Pharmacology, Schulich School of Medicine and Dentistry, and Bone and Joint Institute, University of Western OntarioLondon, ON, Canada; ^5^Faculty of Dentistry, McGill UniversityMontreal, QC, Canada

**Keywords:** ATP, heterologous desensitization, Hill equation, modeling, osteoblast, P2 receptor

## Abstract

Extracellular ATP acts on the P2X family of ligand-gated ion channels and several members of the P2Y family of G protein-coupled receptors to mediate intercellular communication among many cell types including bone-forming osteoblasts. It is known that multiple P2 receptors are expressed on osteoblasts (P2X2,5,6,7 and P2Y1,2,4,6). In the current study, we investigated complex interactions within the P2 receptor network using mathematical modeling. To characterize individual P2 receptors, we extracted data from published studies of overexpressed human and rodent (rat and mouse) receptors and fit their dependencies on ATP concentration using the Hill equation. Next, we examined responses induced by an ensemble of endogenously expressed P2 receptors. Murine osteoblastic cells (MC3T3-E1 cells) were loaded with fluo-4 and stimulated with varying concentrations of extracellular ATP. Elevations in the concentration of cytosolic free calcium ([Ca^2+^]_i_) were monitored by confocal microscopy. Dependence of the calcium response on ATP concentration exhibited a complex pattern that was not explained by the simple addition of individual receptor responses. Fitting the experimental data with a combination of Hill equations from individual receptors revealed that P2Y1 and P2X7 mediated the rise in [Ca^2+^]_i_ at very low and high ATP concentrations, respectively. Interestingly, to describe responses at intermediate ATP concentrations, we had to assume that a receptor with a *K*_1∕2_ in that range (e.g. P2Y4 or P2X5) exerts an inhibitory effect. This study provides new insights into the interactions among individual P2 receptors in producing an ensemble response to extracellular ATP.

## Introduction

Extracellular nucleotides, signaling via P2 receptors, participate in a wide range of biological processes, including neurotransmission, exocrine and endocrine secretion, immune responses, inflammation, pain, and platelet aggregation (Burnstock and Knight, 2004; Orriss et al., 2012). Among the nucleotides, adenosine 5′-triphosphate (ATP) is found in prokaryotes, plants and animals, where it is important for both intracellular and extracellular cell functions. Intracellular ATP is primarily utilized to drive energy-dependent processes, such as active transport (Ataullakhanov and Vitvitsky, 2002). Extracellular ATP has been clearly established to play a role in several biological processes, including the regulation of epithelial cell responses (Schafer et al., 2003), neurotransmission and secretion (Burnstock, 2007), the activation of platelets at sites of vascular injury (Pederson et al., 1999), and bone homeostasis (Weidema et al., 1997; Bowler et al., 2001; Grol et al., 2009; Orriss et al., 2010). The release of ATP can be triggered by various mechanical stimuli, such as shear stress, tension, and hydrostatic pressure (Lazarowski, 2012). Once released into the extracellular milieu, ATP acts on target cells and initiates intracellular signaling through P2 receptors, which are subdivided into the P2X family of ligand-gated ion channels and the P2Y family of G protein-coupled receptors (North, 2002; von Kugelgen, 2006; Orriss et al., 2012). Release of ATP in response to mechanical stimulation, coupled with the presence of multiple P2 receptors on bone cells, has led to the proposal that purinergic signaling plays a key role in skeletal mechanotransduction (Dixon and Sims, 2000).

Currently, seven P2X receptors (P2X1-7) and eight P2Y receptors (P2Y1, P2Y2, P2Y4, P2Y6, P2Y11-14) have been cloned and confirmed in mammals (Gallagher, 2004; Burnstock, 2007). ATP is the sole agonist for the functional homo- or heterotrimeric channels formed by P2X subunits. Upon binding of ATP, these channels change configuration, allowing the entry of Ca^2+^ in addition to the movement of monovalent cations such as Na^+^ and K^+^ (Coddou et al., 2011b). Among P2X receptors, homomeric P2X6 channels were found to be silent as no currents were evoked by ATP when P2X6 was expressed alone (North, 2002; Coddou et al., 2011b). Most G protein-coupled P2Y receptors (P2Y1, P2Y2, P2Y4, P2Y6, P2Y11, P2Y13) are linked to activation of phospholipase C, generation of inositol phosphates and release of Ca^2+^ from intracellular stores (Kennedy et al., 2000; Marteau et al., 2003; White et al., 2003). Depending on the subtype, P2Y receptors can be activated physiologically by ATP, ADP, UTP, UDP, and/or UDP-glucose; however, in the present study, only ATP signaling was addressed. Of the eight P2Y receptors, only P2Y6 and P2Y14 are not activated in the presence of ATP.

Bone forming osteoblasts express at least seven different P2 receptor subtypes (P2X2, P2X5, P2X7, P2Y1, P2Y2, P2Y4, and P2Y6; Orriss et al., 2012). Certain differences in expression in rodent and human tissues can be noted. For instance, the P2Y2 receptor was shown to be strongly expressed on human osteoblasts, whereas P2Y1 receptors were predominant on rat osteoblasts (Gallagher and Buckley, 2002). The functional responses of individual receptors have been studied extensively *in vitro* using heterologous expression techniques to focus on a specific receptor while excluding contributions of others (references in Tables 1, 2). However, less is known about the interactions among endogenously expressed P2 receptors, which determine the overall response to ATP physiologically and pathologically.

**Table 1:** **P2X Receptors: data sources and fitting parameters**.

**P2**	**References**	**Species**	**Cell type**	**Measurement method**	***K_1∕2_* (M)**	***h***	***R*^2^**
P2X1	Allsopp et al., 2011	Human	*Xenopus laevis* oocytes	Electrophysiology	(1.3 ± 0.3) × 10^−6^	0.8 ± 0.2	0.97
	Le et al., 1999	Rat	*Xenopus laevis* oocytes	Electrophysiology	(8.2 ± 0.8) × 10^−7^	4.4 ± 0.9	0.99
P2X2	Roberts et al., 2008	Human	*Xenopus laevis* oocytes	Electrophysiology	(1.2 ± 0.2) × 10^−5^	1.1 ± 0.2	0.99
	Boue-Grabot et al., 2000	Rat	*Xenopus laevis* oocytes	Electrophysiology	(8.2 ± 0.1) × 10^−6^	1.3 ± 0.3	0.99
P2X3	Garcia-Guzman et al., 1997b	Human	*Xenopus laevis* oocytes	Electrophysiology	(5.2 ± 0.6) × 10^−7^	1.1 ± 0.1	0.97
	Lewis et al., 1995	Rat	HEK293	Electrophysiology	(3.4 ± 0.6) × 10^−7^	1.2 ± 0.2	0.99
P2X4	Garcia-Guzman et al., 1997a	Human	*Xenopus laevis* oocytes	Electrophysiology	(7.4 ± 0.4) × 10^−6^	1.4 ± 0.1	0.99
	Garcia-Guzman et al., 1997a	Rat	HEK293	Electrophysiology	(1.1 ± 0.1) × 10^−5^	1.3 ± 0.2	0.99
P2X5	Bo et al., 2003	Human	HEK293	Electrophysiology	(4.5 ± 0.1) × 10^−6^	1.5±0.1	0.99
	Garcia-Guzman et al., 1996	Rodent	HEK293	Electrophysiology	(7.8 ± 0.5) × 10^−6^	1.3 ± 0.1	0.99
P2X6	North, 2002	Human	No currents were evoked by ATP when it was expressed in oocytes or HEK293 cells				
	Roberts et al., 2006	Rat	Rodent P2X6 receptor failed to form functional homotrimeric channels				
P2X7	Roger et al., 2010	Human	HEK293	Electrophysiology	(1.9 ± 0.3) × 10^−3^	2.0 ± 0.4	0.99
	Surprenant et al., 1996	Rat	HEK293	Electrophysiology	(1.3 ± 0.3) × 10^−4^	2.2 ± 0.8	0.97

HEK293-Human Embryonic Kidney 293 cells.

**Table 2:** **P2Y Receptors: data sources and fitting parameters**.

**P2**	**References**	**Species**	**Cell type**	**Measurement method**	***K_1∕2_* (M)**	***h***	***R*^2^**
P2Y1	Palmer et al., 1998	Human	1321N1	Cytosolic Ca2+ measurements	(3.0 ± 0.3) × 10^−7^	0.9 ± 0.1	0.99
	Vohringer et al., 2000	Rat	HEK293	Cytosolic Ca2+ measurements	(3 ± 1) × 10^−7^	0.5 ± 0.1	0.97
P2Y2	Nicholas et al., 1996	Human	1321N1	IP3 accumulation	(2.0 ± 0.4) × 10^−7^	1.2 ± 0.3	0.98
	Wildman et al., 2003	Rat	*Xenopus laevis* oocytes	Electrophysiology	(2.9 ± 0.4) × 10^−6^	1.4 ± 0.2	0.99
P2Y4	Nicholas et al., 1996	Human	1321N1	IP3 accumulation	(2.9 ± 0.2) × 10^−5^	1.3 ± 0.1	0.99
	Wildman et al., 2003	Rat	*Xenopus laevis* oocytes	Electrophysiology	(1.2 ± 0.2) × 10^−6^	1.0 ± 0.2	0.99
P2Y6	Nicholas et al., 1996	Human	Human P2Y6 has not shown significant sensitivity to ATP				
	Lazarowski et al., 2001	Mouse	Mouse P2Y6 has not shown significant sensitivity to ATP				
P2Y11	Qi et al., 2001	Human	1321N1	IP3 accumulation	(2.5 ± 0.1) × 10^−6^	1.1 ± 0.1	0.99
	von Kugelgen, 2006	Rodent	In rat and mouse, P2Y11 transcripts have not been found				
P2Y12	von Kugelgen, 2006	Human	Human P2Y12 receptor has not shown significant sensitivity to ATP				
	Ennion et al., 2004	Bovine	Chromaffin cells	Electrophysiology	(3.7 ± 0.7) × 10^−6^	0.8 ± 0.1	0.99
P2Y13	Marteau et al., 2003	Human	1321N1	IP3 accumulation	(5 ± 2) × 10^−6^	1.2 ± 0.4	0.97
	Fumagalli et al., 2004	Rat	Rat P2Y13 has not shown significant sensitivity to ATP				
P2Y14	Chambers et al., 2000	Human	Human P2Y14 has not shown significant sensitivity to ATP				
	von Kugelgen, 2006	Rodent	Rat and mouse P2Y14 has not shown significant sensitivity to ATP				

1321N1 human astrocytoma cells; HEK293 human embryonic kidney 293 cells; IP_3_ inositol 1,4,5-trisphosphate.

The goal of this study was to use previously published ATP dose-dependence data for individual P2 receptors to gain insight into how these receptors might interact when expressed together in an endogenous network of P2 receptors present in the osteoblastic cell line MC3T3-E1. To do this, we first summarized published data on the ATP concentration dependency of individual P2 receptors. We then performed detailed analysis of the dependence of calcium responses in osteoblastic cells on extracellular ATP concentration. Finally, we modeled the potential contribution of individual receptors to ATP responses in osteoblastic cells endogenously expressing an ensemble of P2 receptor subtypes.

## Materials and methods

### Cells and cell culture

The MC3T3-E1 osteoblast-like cell line, a non-transformed clonal cell line originally established from newborn mouse calvaria (Sudo et al., 1983), was from the American Type Culture Collection (Rockville, MD, USA). Subclone 4 of MC3T3-E1 cells was selected because these cells exhibit properties of osteoblasts, including elevation of cyclic AMP in response to parathyroid hormone. expression of transcripts for Runx2, bone sialoprotein, and osteocalcin, and formation of mineralized nodules (Wang et al., 1999). Importantly, MC3T3-E1 cells endogenously express multiple subtypes of P2X and P2Y receptors (Gartland et al., 2012; Grol et al., 2013; Xing et al., 2014). Functionally, extracellular nucleotides acting through P2 receptors on MC3T3-E1 cells have been reported to stimulate: cell proliferation (Shimegi, 1996); prostaglandin release (Genetos et al., 2005); cell metabolism (Grol et al., 2012); Ca^2+^-NFATc1 signaling (Grol et al., 2013); and the Wnt/β-catenin signaling pathway (Grol et al., 2016). Thus, the MC3T3-E1 cell line is an excellent system for examining interactions among endogenously expressed P2 receptors in a physiologically relevant cell type. MC3T3-E1 cells were maintained at 37°C and 5% CO_2_ in α-minimum essential medium, supplemented with 10% fetal bovine serum and 1% antibiotic solution (10,000 U/mL penicillin, 10,000 μg/mL streptomycin, and 25 μg/mL amphotericin B). All cell culture reagents were from Life Technologies Inc. (Burlington, ON, Canada).

### Fluorescence measurement of cytosolic free calcium concentration ([ca^2+^]_i_)

[Ca^2+^]_i_ measurements used in the present study were unpublished single-cell data from our previous work (Grol et al., 2013). Briefly, MC3T3-E1 cells were plated at a density of 1.5 × 10^4^ cells/cm^2^ on 35-mm glass-bottomed dishes (MatTek Corporation, Ashland, MA, USA) in culture medium. After 2 days, cells were placed in serum-free medium and incubated overnight. On the day of the experiment, cells were loaded with the Ca^2+^-sensitive dye fluo-4 by incubation with fluo-4-AM (2 μg/mL) and 0.1% Pluronic F-127 (Molecular Probes, Life Technologies) for 30–45 min at 37°C and 5% CO_2_. Medium was then replaced with HEPES-buffered, bicarbonate-free α-MEM supplemented with 1% antibiotic solution, and cells were observed by live-cell confocal microscopy (model LSM 510; Carl Zeiss Inc., Jena, Germany) at ~28°C using a Plan-Apochromat 40 × objective (1.2 NA; Carl Zeiss Inc.) with 488 nm Ar^+^ ion laser excitation. Images were captured at 500–550 emission every 500 ms in time-lapse mode. Fluorescence intensity was analyzed using LSM 510 software. Baseline fluorescence intensity in different experiments varied between 23 ± 7 and 78 ± 43 FU. Within individual experiments basal fluorescence intensity between different experimental conditions varied by 17 ± 8%. [Ca^2+^]_i_ in individual cells was quantified as normalized fluorescence intensity using the formula (F/F_o_)−1, where F was fluorescence intensity and F_o_ was the baseline fluorescence observed prior to application of adenosine 5′-triphosphate disodium salt (ATP, Sigma-Aldrich, St. Louis, MO, USA) to the bath solution.

### Data analysis

Experimental data are presented as representative traces or means ± standard deviation (SD) when the responses of individual cells within a single experiment were analyzed or standard error of the mean (SEM) when the results of a number of independent experiments were analyzed. MATLAB (The MathWorks Inc., Natick, MA, 2000) was used to extract and analyze the calcium response data and to calculate the average peak amplitude, duration and area under the curve (Supplementary Matlab code 1). The outliers were identified as points outside of mean ± 2 SD within the group of cells exposed to the same ATP concentration within the same experiment. Between 25% (for low ATP concentrations) and 10% (for high ATP concentrations) of cells were removed from analysis as outliers. Data within an individual experiment was normalized to maximum in each corresponding parameter to account for biological and experimental variability. Comparative analyses of outcomes using raw data and normalized data demonstrated that only for the amplitude dose dependence was statistical significance lost when raw data were used, due to higher variability in this parameter. Differences were assessed by ANOVA for multiple group comparisons, followed by Tukey post-test, and accepted as statistically significant at *p* < 0.05. To examine the contribution of individual receptors to the overall calcium response, we used a linear combination approach, in which the complex response was assumed to arise from the sum of responses of individual receptors multiplied by the respective coefficients. We defined the set of basic functions as the concentration dependences of individual receptors obtained from literature analysis, and assumed that a complex ATP-dependence is a sum of basic functions executed with certain weight (coefficients): *f(x)* = *a*_1_*f*_1_*(x)* + *a*_2_*f*_2_*(x)* + …+ *a*_*n*_*f*_*n*_*(x)*, where *f*_*i*_ (*i* = 1, 2, …n) are the ATP concentration dependencies of individual receptors and *a*_*i*_ (*i* = 1, 2, …n) are coefficients that roughly correspond to the efficiency of engagement of different receptors as well as generally stimulatory (positive contribution) or inhibitory (negative contribution) of individual functions (receptors) to the overall response. The coefficients in the linear combination function were then fitted numerically.

### Fitting methods

The data points of each receptor were extracted from indicated published sources using software DataThief (B. Tummers, DataThief III. 2006). These data were then fit to the Hill equation using a non-linear least squares regression program in MATLAB (Supplementary Matlab code 2). The Hill coefficient, half maximum value and linear combination coefficients (Supplementary Matlab code 3) were obtained from the fitting, and are presented as best fit value ± 95% confidence interval.

## Results

### Characteristics of individual P2 receptors

**Figure 1:**
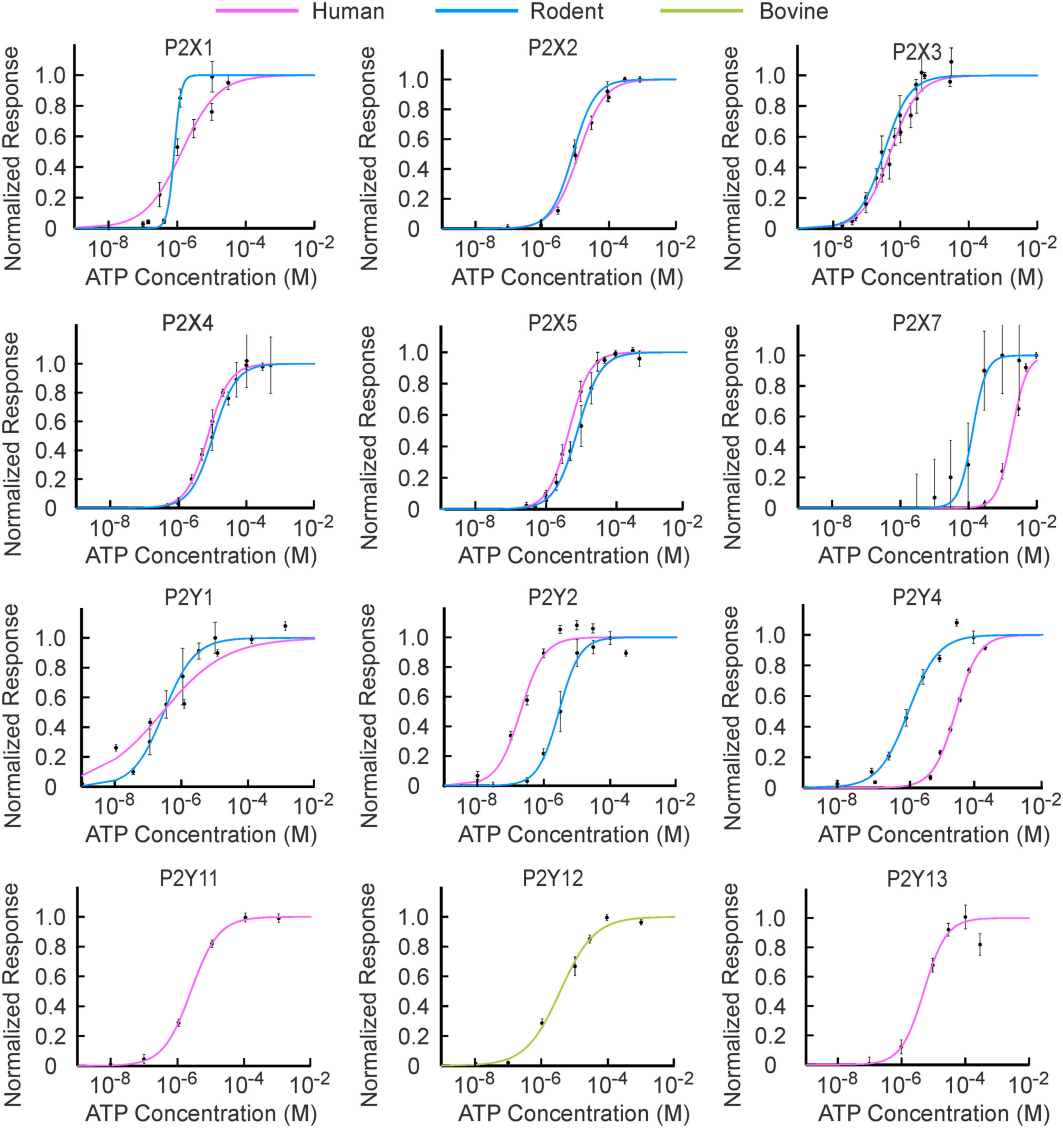
**[ATP] sensitivity of P2X and P2Y receptor families**. The experimental data points for human and rodent P2 receptors denoted by dots were re-plotted from papers indicated in Tables 1, 2. P2Y11 transcripts have not been found in rodents; therefore, only human P2Y11 was used. The experimental data for [ATP] dependence of human and rodent P2Y12 and rodent P2Y13 were not found in the literature, therefore bovine P2Y12 and human P2Y13 were used. The data were normalized to the maximal response. [ATP] dependence for each receptor was fit with the Hill equation with R^2^ > 0.95, and shown in pink for human, blue for rodent, and green for bovine receptors.

Using previously published data, we first explored the dependence on ATP concentration of responses induced by each P2 receptor. Within each of the studies, an individual P2X (Table 1) or P2Y (Table 2) receptor cDNA sequence was isolated and overexpressed in various cell-types and their dependency on ATP concentration was measured. The data from the concentration dependence curves obtained in each study were extracted, normalized to the maximal response and fitted to the Hill equation (Figure 1; Evans et al., 1995):

$$\begin{array}{l} \theta =\frac{L^{h}}{K_{1∕2}^{h}+L^{h}}, \end{array}$$

where θ is the fraction of activated receptors; L represents ligand concentration; *K*_1∕2_ is the ligand concentration at half maximal response, and *h* is the Hill coefficient, describing cooperativity (Figure 1, Tables 1, 2).

**Figure 2:**
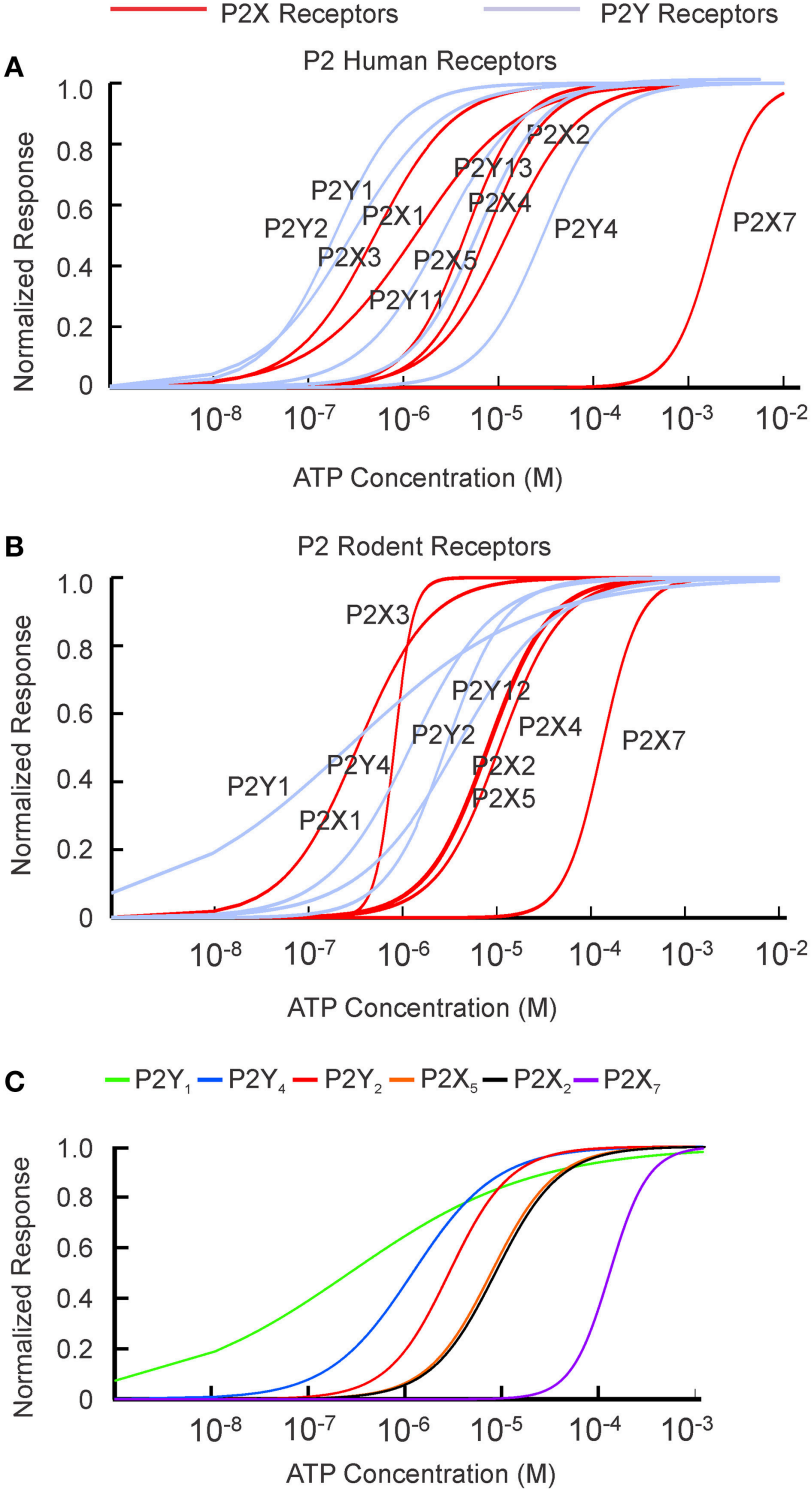
**P2 receptors are activated by a wide range of ATP concentrations**. ATP concentration dependence obtained from the fitting of published data (Tables 1, 2) to the Hill equation for P2X receptors (in red) and P2Y receptors (in light purple) were plotted for human **(A)** and rodent **(B)** receptors. **(C)** P2 receptors known to be expressed on osteoblasts.

The [ATP] dependence of rodent and human receptors overlapped for P2X2-5 receptors. Rodent and human P2X1 and P2Y1 receptors demonstrated similar *K*_1∕2_ but different cooperativity *h*, which was higher for rodent P2X1 and P2Y1 receptors. P2Y2, P2Y4, and P2X7 exhibited a difference in *K*_1∕2_ between human and rodent receptors. Rodent P2X7 and P2Y4 were more sensitive to [ATP], whereas P2Y2 was less sensitive compared to their human counterparts (Figure 1, Tables 1, 2). When plotted together, the concentration dependence curves for both human (Figure 2A) and rodent (Figure 2B) receptors covered a wide range of [ATP]; however, rodent receptors were positioned in a lower concentration range from 10^−9^ to 10^−3^ M, which was shifted to the right by approximately an order of magnitude for human receptors, 10^−8^ to 10^−2^ M. To assess the contributions of individual P2 receptors to ATP-induced calcium responses in osteoblasts, we plotted [ATP]-dependencies of the known ATP-responsive P2 receptors expressed by osteoblastic cells P2X2,5,7 and P2Y1,2,4 (Figure 2C).

### Experimental data for the dependence of ca^2+^ responses on ATP concentration

**Figure 3:**
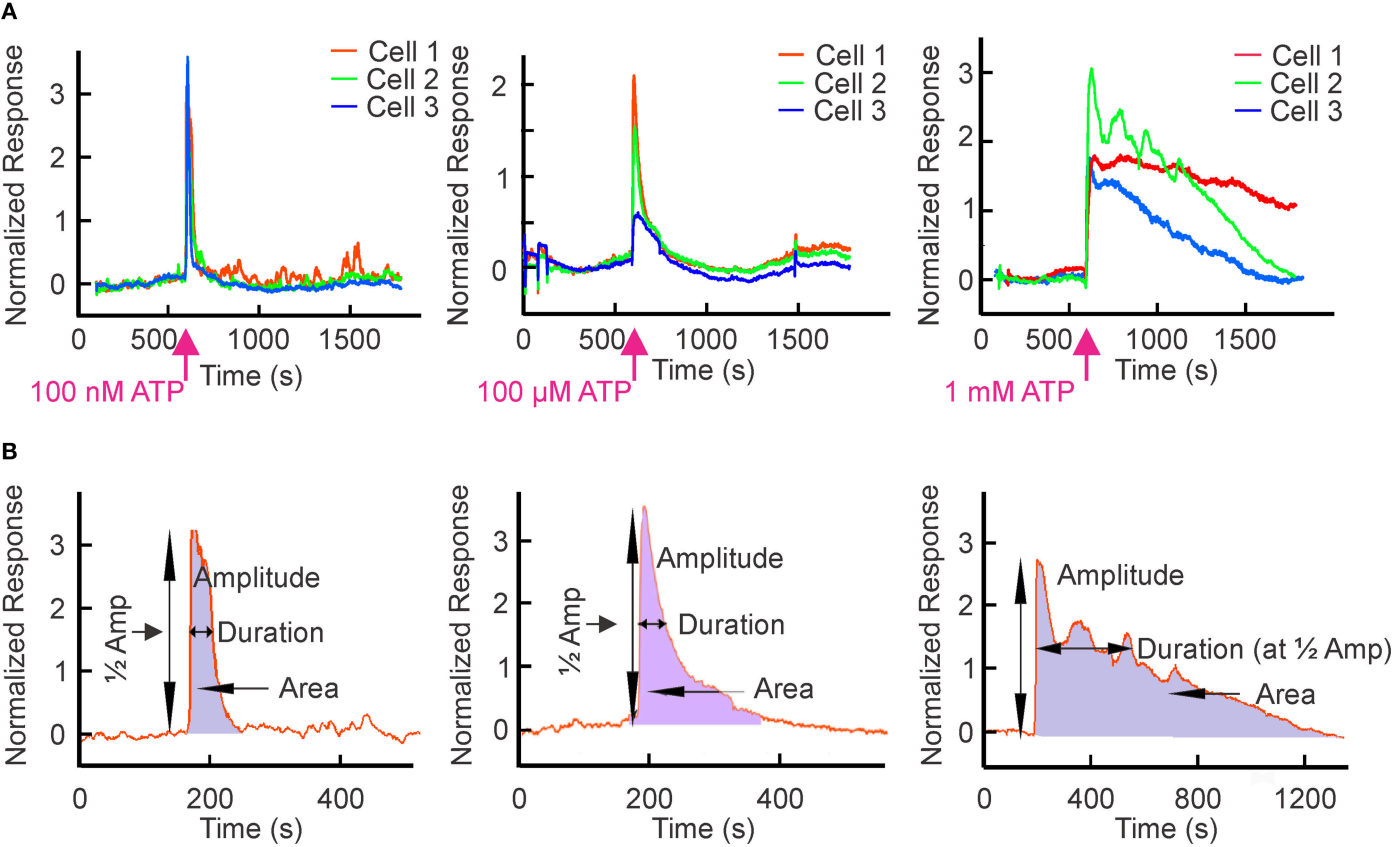
**ATP-induced Ca^**2+**^ responses in osteoblastic cells**. MC3T3-E1 cells were loaded with the Ca^2+^ sensitive dye fluo-4 and changes in [Ca^2+^]_i_ in response to bath application of ATP were monitored by confocal microscopy. [Ca^2+^]_i_ in individual cells was normalized as (F/F_0_)−1, where F was fluorescence intensity and F_0_ was the baseline fluorescence observed prior to addition of ATP. **(A)** Representative calcium responses from osteoblasts exposed to 100 nM, 100 μM or 1 mM ATP at the time indicated by the arrow. **(B)** The schematics illustrate parameters used to analyse the calcium response curves. Peak amplitude was quantified as the maximal rise in [Ca^2+^]_i_. Duration of the response was quantified as the width at the half maximum. The amount of calcium released was calculated as the area under the curve by taking the integral of the curve.

We used the murine osteoblast-like cell line MC3T3-E1, which endogenously expresses an ensemble of P2X and P2Y receptors (Grol et al., 2013). Cells were loaded with the calcium-sensitive fluorescent probe fluo-4 and ATP-induced changes in [Ca^2+^]_i_ were assessed. Extracellular ATP from 1 nM to 1 mM induced transient elevation of [Ca^2+^]_i_ with distinctive patterns at different concentrations (Figure 3A). We characterized these ATP-induced calcium responses by determining: (i) the peak amplitude; (ii) the width at half maximum, which reflects the duration of the calcium response; and (iii) the area under the curve, which reflects the amount of calcium released over time (Figure 3B). We analyzed individual responses of 21–61 cells to 8–11 different concentrations of ATP in 8 independent experiments (2607 cells in total).

### An ATP sensitivity threshold was generated by P2Y1 (with potential involvement of P2Y4) at low concentrations of ATP

**Figure 4:**
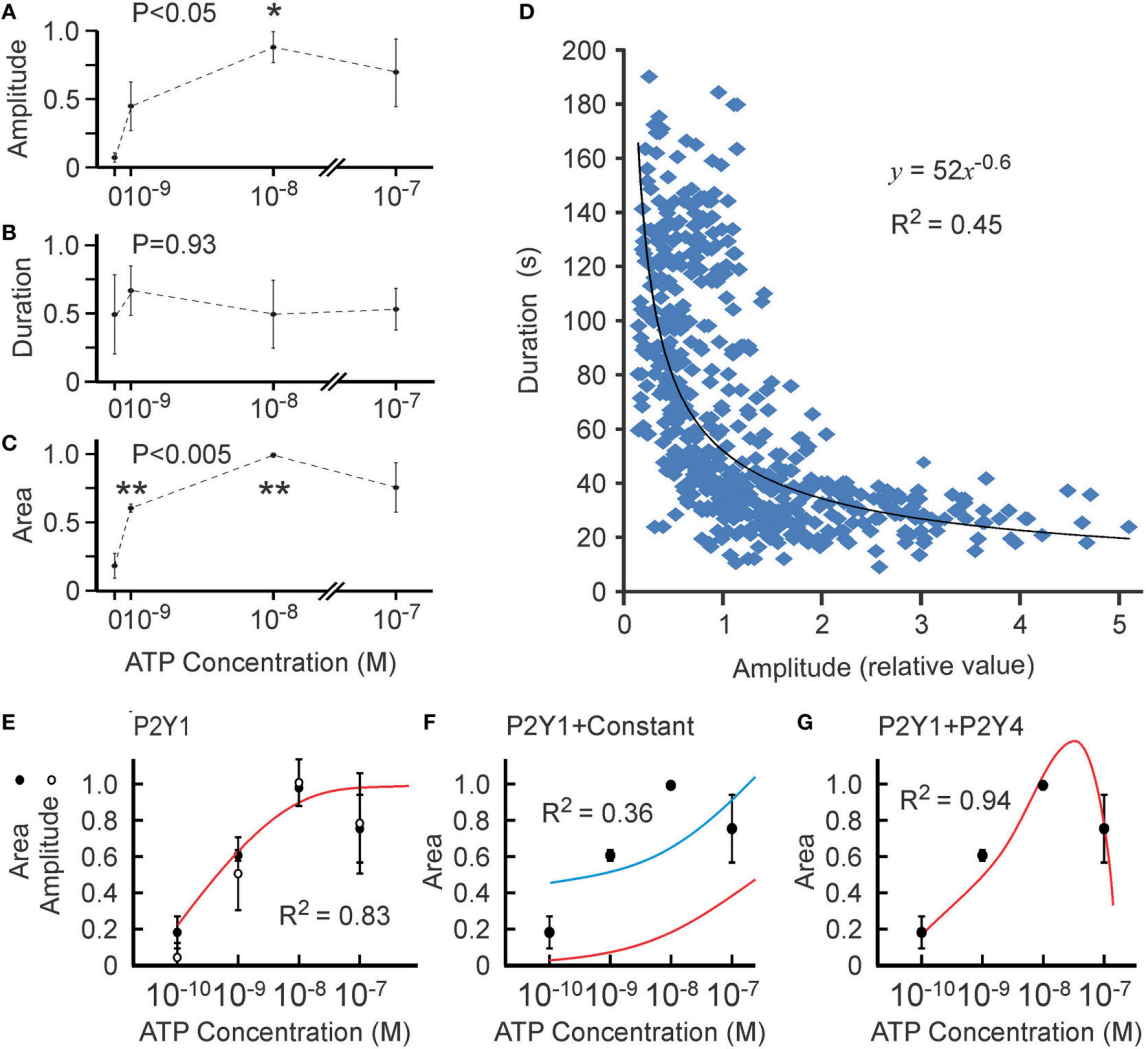
**Ca^**2+**^ responses induced by low [ATP]**. Changes in [Ca^2+^]_i_ induced by bath application of vehicle (0) or 10^−9^–10^−7^ M ATP to MC3T3-E1 cells loaded with fluo-4 were analyzed. **(A)** [ATP] dependence of average peak amplitude. **(B)** [ATP] dependence of average response duration. **(C)** [ATP] dependence of average area under the curve. For **(A–C)**, data are means ± SEM, normalized to the maximal amplitude **(A)**, duration **(B)** or area under the curve **(C)** (maximal response = 1) within an experiment, *n* = 3 independent experiments, 17–35 cells per condition in each experiment. The differences were analyzed by ANOVA (*P*-value given on the top of the graph) followed by Tukey post-test, ^*^p < 0.05; ^**^p < 0.01 compared to vehicle (0). **(D)** The correlation between the amplitude and duration of Ca^2+^ responses was examined in 563 individual cells exposed to 10^−9^–10^−7^ M ATP. **(E)** Similar best fit for [ATP] dependence for amplitude (white circles) and area (black circles) of Ca^2+^ responses. **(F)** The [ATP] dependence for the area was fit with the Hill equation of P2Y1 based on published data yielding no acceptable fit (red curve). The Hill equation of P2Y1 offset by a constant β is shown as the blue curve (*ky1* = 1 ± 2, β = 0.4 ± 0.6, *R*^2^ = 0.36). **(G)** The [ATP] dependence for the area under Ca^2+^ responses was fit with the linear combination of P2Y1 and P2Y4 functions (*ky1* = 7 ± 1, *ky4* = −25 ± 8, *R*^2^ = 0.94).

We first examined the dose dependence of calcium responses at very low ATP concentrations (10^−9^–10^−7^ M). In these experiments, application of vehicle commonly induced low-level responses likely attributable to mechanical stimulation of osteoblasts due to fluid shear. Therefore, we chose three experiments in which the cell responses to vehicle were absent or low and analyzed them separately for the responses to ATP in the range of 10^−9^–10^−7^ M (Figure 4). We found that the average amplitude of calcium responses exhibited a trend to increase at 10^−9^ M [ATP] and was significantly higher than in vehicle-treated cells at 10^−8^ M [ATP] (Figure 4A). The average duration of the calcium signal did not change at these concentrations (Figure 4B). The average area under the curve increased significantly at 10^−9^ M [ATP] compared to vehicle and increased further at 10^−8^ compared to 10^−9^ M [ATP] (Figure 4C). Interestingly, variation in the amplitude of the calcium response (reflected by the size of the error bars) was greater than in the amount of calcium released estimated as the area under the curve. When we compared the amplitude and duration of calcium responses in individual cells, we found a significant negative correlation between these parameters (Figure 4D), suggesting that amplitude and duration are not independent parameters, but rather exhibit a complex non-linear correlation represented by an exponential regression line. The best fit for amplitude [*K*_1∕2_ = (7 ± 9) × 10^−10^ M, *h* = 1 ± 1, *R*^2^ = 0.87] and area [*K*_1∕2_ = (5 ± 7) × 10^−10^ M, *h* = 0.8 ± 0.5, *R*^2^ = 0.83] curves were similar (Figure 4E).

Since P2Y1 is the most sensitive ATP receptor, we first assumed that P2Y1 is the only receptor contributing to the calcium response in the 10^−9^–10^−7^ M range, and fit the area curve with the Hill equation of P2Y1. However, the sensitivity of P2Y1 observed in overexpression experiments (Table 2) was not sufficient to describe the responses to ATP at 10^−9^–10^−8^ M observed in MC3T3-E1 cells with an endogenous ensemble of P2 receptors (Figure 4F, red curve). One strategy to obtain a better fit was to shift the curve up, assuming a constant extraneous contribution to the response (such as mechanical perturbation of the cell induced by application of the test solution: Grol et al., 2013). The best fit available had *R*^2^ = 0.36 and required a non-specific contribution of β = 0.43 (Figure 4F, blue curve). As another possibility, we considered simultaneous activation of P2Y1 and P2Y4, modeled as a linear combination of these receptors: θ(*x*) = ky1 × P2Y1 + ky4 × P2Y4. Interestingly, the best resulting fit had an *R*^2^ = 0.94 (Figure 4G) and required the contribution of P2Y4 to be inhibitory.

### A second threshold was generated by P2X7 at high concentrations of ATP

**Figure 5:**
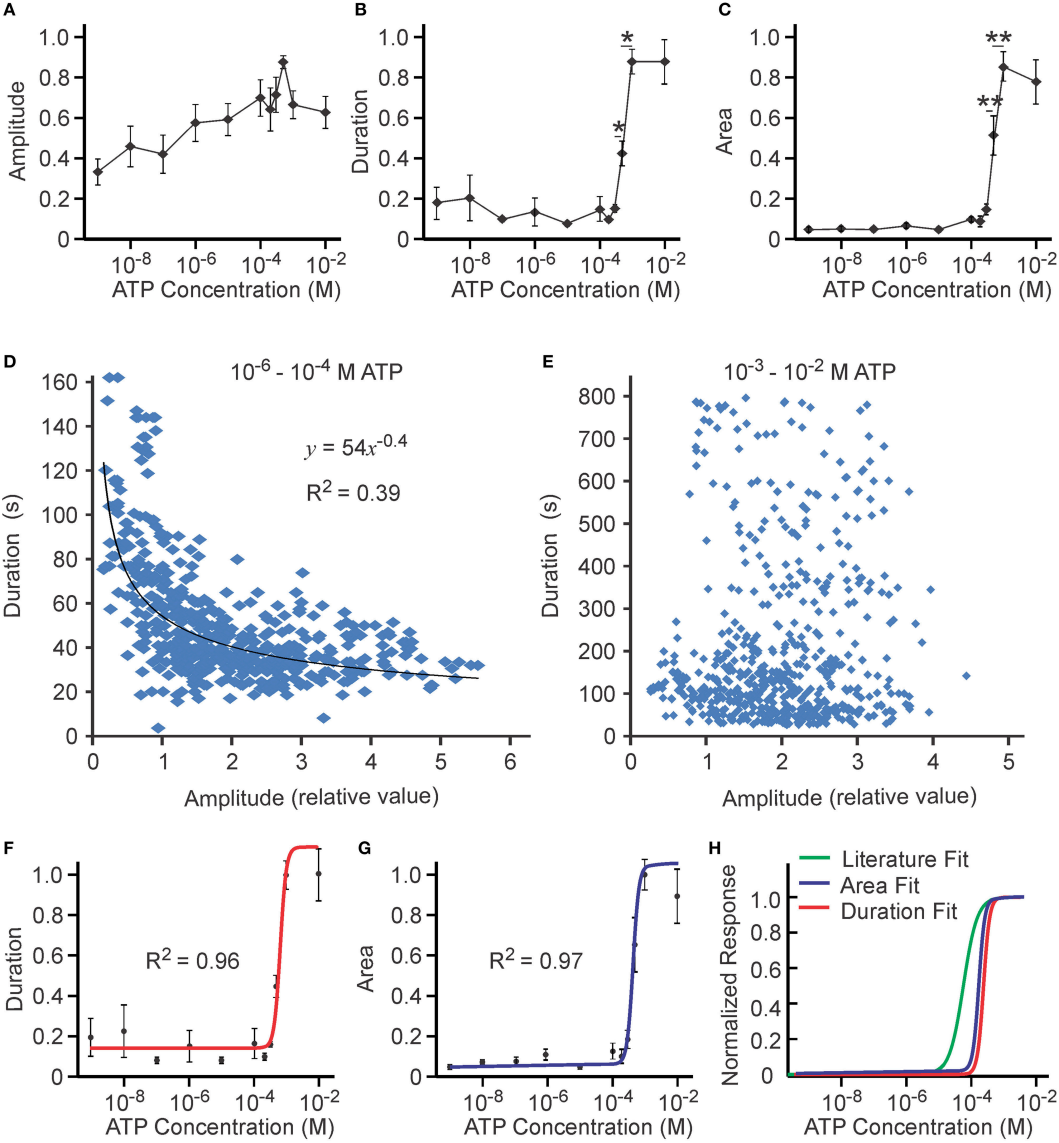
**Ca^**2+**^ responses induced by high [ATP]**. Changes in [Ca^2+^]_i_ induced by bath application of 10^−9^–10^−2^ M ATP to MC3T3-E1 cells loaded with fluo-4 were analyzed. **(A)** [ATP] dependence of average peak amplitude. **(B)** [ATP] dependence of average response duration. **(C)** [ATP] dependence of average area under calcium response. For **(A–C)** data are means ± SEM, *n* = 8 independent experiments, 11–55 cells per condition in each experiment. Differences (indicated by horizontal lines) were analyzed by ANOVA and were significant for **(B,C)**, *p* < 0.0001. Selected statistically significant differences are shown with asterisks that demonstrate significant increases with an increase in applied [ATP] assessed by Tukey post-test (^*^p < 0.05, ^**^p < 0.01). **(D,E)** The correlation between the amplitude and duration of Ca^2+^ responses was examined in 520 individual cells exposed to 10^−6^–10^−4^ M ATP **(D)** and in 588 individual cells exposed to 10^−3^–10^−2^ M ATP **(E)**. **(F)** The [ATP] dependence for the duration of calcium response was fit with the Hill equation with *K*_1∕2_ = (6.0 ± 0.6) × 10^−4^ M and *h* = 4 ± 1 and an offset of β = 0.13 ± 0.03. **(G)** The [ATP] dependence for the area under calcium response was fit with the Hill equation with *K*_1∕2_ = (4.6 ± 0.3) × 10^−4^ M and *h* = 4 ± 1 with an offset of β = 0.05 ± 0.02. **(H)** Comparison of published P2X7 fit (green line) obtained for the overexpressed receptor [*K*_1∕2_ = (1.3 ± 0.3) × 10^−4^ M and *h* = 2.2 ± 0.8; (Surprenant et al., 1996)] to the fits for average duration (red line, same as in **F**) and average area (blue line, same as in **G**) observed in our experimental data.

We next examined ATP concentration dependence over 10^−9^–10^−2^ M in eight independent experiments using MC3T3-E1 cells. When the concentration of ATP was increased, an increase in the amplitude of the calcium response was observed (Figure 5A). The duration of the calcium signal increased only when [ATP] exceeded 10^−4^ M, a concentration at which P2X7 is activated (Figure 5B). The concentration dependence for the area under the curve (characterizing the amount of calcium released during the response) revealed the presence of a second threshold—a sharp increase in the area between 10^−4^ and 10^−3^ M ATP (Figure 5C). Similar to the low ATP concentration range, variability in the area under the curve was noticeably smaller when compared to variability in duration or amplitude. Though the negative correlation between duration and amplitude observed at low ATP was still significant at 10^−6^–10^−4^ M [ATP] (Figure 5D), it was completely lost at 10^−3^–10^−2^ M [ATP] (Figure 5E). The best fits for the duration curve (*R*^2^ = 0.96, Figure 5F) and the area curve (*R*^2^ = 0.97, Figure 5G) exhibited similar *K*_1∕2_ of (6.0 ± 0.6) × 10^−4^ M and (4.6 ± 0.3) × 10^−4^ M, respectively. The same cooperativity of *h* = 4 ± 1 and *h* = 4 ± 1 was also observed for both. Whereas the P2X7 fit obtained for the overexpressed receptor was similar to our study, we observed both higher *K*_1∕2_ and higher cooperativity in our experimental data (Figure 5H).

### Complex dependence of amplitude on concentration in the intermediate range of [ATP]

**Figure 6:**
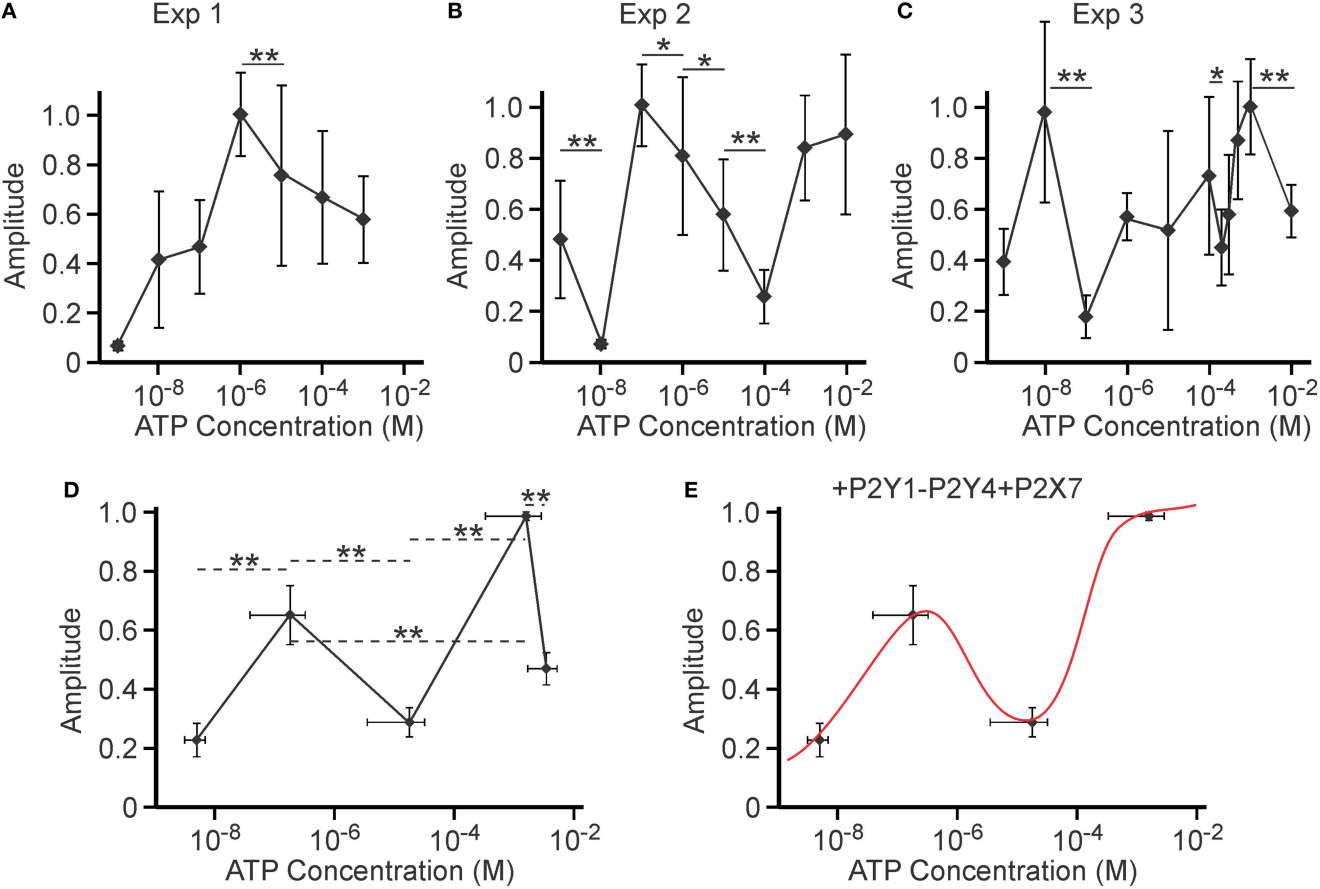
**Potential inhibitory role of a P2 receptor activated at intermediate [ATP]**. Amplitudes of Ca^2+^ responses induced by bath application of 10^−9^–10^−2^ M ATP to MC3T3-E1 cells loaded with fluo-4 were analyzed. **(A–C)** The examples of [ATP] dependencies of the peak amplitude of calcium response observed in three independent experiments. Data are means ± SEM, normalized to the maximal response (maximal response = 1) within an experiment, *n* = 19–25 cells/concentration for **(A)**, 11–27 cells/concentration for **(B)**, 17–31 cells/concentration for **(C)**. Differences (indicated by horizontal lines) were analyzed by ANOVA and were significant for all cases, with *p* < 0.0001. Selected statistically significant differences are shown with asterisks that demonstrate significant decreases in amplitude with an increase in applied [ATP] assessed by Tukey post-test (^*^p < 0.05, ^**^p < 0.01). **(D)** Eight experiments were analyzed for ATP concentrations and relative amplitudes of (i) the initial low response, (ii) the first peak, (iii) the lowest value between the peaks, (iv) the second peak, and (v) the lowest value after the second peak. Means ± SEM for [ATP] and relative amplitudes were plotted. Differences (indicated by horizontal dashed lines) were analyzed by ANOVA (*p* < 0.0001), followed by Tukey post-test; ^**^p < 0.01. **(E)** The resulting concentration dependence was fit with the linear combination of P2Y1, P2Y4, and P2X7 functions (*ky1* = 1.8 ± 0.4; *ky4* = −1.4±0.4; *kx7* = 0.6 ± 0.2, *R*^2^ = 0.99).

During the analysis, we noticed that the ATP concentration dependence for amplitude of calcium responses averaged for 8 independent experiments was described by a continuously increasing curve (Figure 5A). However, such a curve was not representative of any of the 8 individual experiments, leading us to conclude that, under this circumstance, average cannot be used as a measure of central tendency for the underlying ATP concentration dependence. In fact, within each individual experiment the [ATP]-dependence of amplitude averaged for 11–55 distinct cells exhibited significant non-linearity (3 examples are given in Figures 6A–C). Specifically, we observed that the amplitude decreased with increasing [ATP] and then increased again, resulting in peaks in the intermediate range of [ATP]. In one out of eight independent experiments, there was only one peak (Figure 6A); whereas, in the 7 others, there were at least 2 such peaks (Figures 6B,C). Since the position of these peaks within the ATP concentration dependence curves varied, we analyzed each experiment for the ATP concentrations at which we observed (*a*) the initial low response, (*b*) the first peak, (*c*) the lowest value between the peaks, (*d*) the second peak, and (*e*) the lowest value after the second peak, as well as the relative amplitudes at each point (Figure 6D). We found that such analyses revealed 2 distinct peaks in amplitude for the calcium response—a lower peak at 180 ± 140 nM ATP and a higher peak at 1.6 ± 1.2 mM ATP. We removed the highest [ATP] point from the concentration-dependence due to potential non-specific effects of 10 mM ATP (Grol et al., 2013). The resulting relationship was modeled with a linear combination of published [ATP] dependencies for P2Y1, P2Y4, and P2X7: θ(x) = ky1 × P2Y1 + ky4 × P2Y4 + kx7 × P2X7. An excellent fit (*R*^2^ = 0.99) was given by the following linear combination of constants: *ky1* = 1.8 ± 0.4; *ky4* = −1.4±0.4; *kx7* = 0.6 ± 0.2 (note that the contribution from P2Y4 was again inhibitory; Figure 6E). This curve could also be successfully described by a linear combination of P2Y1 and P2X7 with inhibitory P2X5 (*ky1* = 1.5 ± 0.3, *kx5* = −1.4±0.4, *kx7* = 0.91 ± 0.2, *R*^2^ = 0.99) or inhibitory P2X2 (*ky1* = 1.5 ± 0.3, *kx2* = −1.4±0.4, *kx7* = 0.95 ± 0.3, *R*^2^ = 0.99).

## Discussion

In this study, we examined the interactions among P2 receptors endogenously expressed by mouse osteoblastic cells and how such interactions shape the profiles of transient Ca^2+^ elevations induced by ATP over a wide range of concentrations. Experimentally, we examined Ca^2+^ elevations in response to 10^−9^–10^−2^ M ATP and observed complex dependence on [ATP] for amplitude, duration and area under the curve. In particular, the area under the curve (reflecting the amount of calcium released) demonstrated the presence of two thresholds—the first at approximately 1 nM ATP and the second at approximately 1 mM ATP. At low [ATP], an increase in the amplitude of Ca^2+^ responses created a sensitivity threshold in the ATP concentration dependence. At high [ATP], an increase in the duration of response coincided with a second threshold. Finally, the amplitude of calcium responses demonstrated a complex two-peak pattern suggesting the presence of desensitization at intermediate concentrations of ATP. To explain these complex trends, we used published data for [ATP] dependencies of individual P2 receptors. We found that a linear combination of different receptor functions described calcium responses over a wide range of [ATP], if one of the receptors activated by mid-range concentrations of ATP was assumed to exhibit an inhibitory effect on the ATP-induced calcium response.

### Sensitivity threshold at low concentrations of ATP

At low ATP concentrations from 1 to 100 nM, P2Y1 is the predominant rodent receptor responsible for Ca^2+^ elevations. Published data on the P2Y1 receptor reported a *K*_1∕2_ of 50–200 nM ATP (Palmer et al., 1998; Vohringer et al., 2000); whereas, our experimental data on osteoblastic cells requires P2Y1 to be more sensitive to ATP (*K*_1∕2_ ~ 1 nM). We could partially account for this discrepancy by assuming that mechanical perturbation contributed to the calcium response, as previously described (Grol et al., 2013); however, the fit obtained with such an assumption was not very good (*R*^2^ = 0.36). On the other hand, when we considered the simultaneous activation of P2Y1 and P2Y4, we achieved an increase in the apparent sensitivity of P2Y1 to 1 nM ATP. Interestingly, the increased sensitivity of P2Y1 was only possible if we assumed an inhibitory effect of P2Y4 activation. P2Y4-mediated inhibition permits the situation where the response to P2Y1 stimulation is limited to an apparent maximum, while the actual maximal response is greater. As a result, the apparent *K*_1∕2_ for P2Y1 is shifted to the left. Although it is known that allosteric regulators can affect the apparent *K*_1∕2_ of receptor-ligand interactions (Hulme and Trevethick, 2010), we describe a mechanism in which the function of an individual receptor is not affected, but the simultaneous activation of stimulatory and inhibitory pathways changes the apparent *K*_1∕2_. Importantly, such heterologous desensitization allows for generation of a steep threshold, while at the same time limiting the maximal level of stimulation. This phenomenon may be relevant to diverse systems in which sharp response thresholds are observed and multiple receptors are activated by the same or similar ligands, such as eicosanoid signaling (Rundhaug et al., 2011), odor recognition (Dewan et al., 2013), and neurotransmission by nicotinic receptors (Exley and Cragg, 2008).

### P2X7 is responsible for responses to ATP at high concentrations

Our study suggests that cell responses to high [ATP] above 200 μM reflect activation of the P2X7 receptor. It was previously known that only activation of P2X7 results in prolonged elevation of calcium (Virginio et al., 1999; Naemsch et al., 2001; Grol et al., 2012; Jiang et al., 2013). In P2X7 knockout mice, the Ca^2+^ response is transient, similar to the response induced by low [ATP] (Grol et al., 2013). In the present study, we found that duration of calcium responses was not dependent on [ATP] at concentrations less than 10^−4^ M, but strongly increased at higher concentrations with *K*_1∕2_ of 600 ± 120 μM. We also found that the second threshold in area under the curve mirrored that of the increase in duration. In our study, the observed sensitivity of the P2X7 receptor was less than that reported for overexpressed receptors, whereas the cooperativity was greater (Surprenant et al., 1996; Roger et al., 2010). Millimolar levels of ATP represent the normal intracellular concentration range for ATP (Ataullakhanov and Vitvitsky, 2002). Therefore, extracellular ATP in the millimolar range likely occurs following cell damage, or in response to vigorous mechanical stimuli. It is well-known that bone adapts to mechanical loading by increasing its mass. In this regard, activation of P2X7 receptors by extracellular ATP in the millimolar range has been proposed to stimulate anabolic responses in bone (Ke et al., 2003; Li et al., 2005; Panupinthu et al., 2008; Grol et al., 2013, 2016). Therefore, we propose that the second, P2X7-mediated threshold in calcium responses to ATP provides an anabolic signal following robust mechanical stimulation as well as during the early stages of wound healing following trauma.

### Inhibitory contribution of a P2 receptor with mid-range sensitivity to ATP

We found that the amplitude of P2 receptor-induced calcium responses exhibited an unexpected dependence on ATP concentration. Specifically, a significant decrease in peak amplitude could be observed with increases in [ATP] at mid-range concentrations of 10^−7^–10^−4^ M. Agonist concentration dependencies that could not be simply explained by known P2 receptor characteristics have been described previously for neuronal cells (Patel et al., 2001) as well as taste buds cells (Fedorov et al., 2007). We found that, to describe this behavior using only a linear combination of known receptors, it was necessary to assume that one or more of the receptors activated by mid-range ATP concentrations contributes negatively to the overall response.

Although, none of the P2 receptors expressed individually demonstrates inverse agonism in response to ATP, several reasons for observing such behavior in naïve cells can be suggested. First, different P2 receptors can form heteromers with physiological and pharmacological properties distinct from homomeric receptors (Surprenant et al., 2000; Coddou et al., 2011b; Compan et al., 2012). Second, the availability of different receptors can be regulated in complex manner. For example, it was reported that P2X4 receptors undergo rapid internalization in olfactory neurons, whereas P2X2 are not regulated by translocation (Bobanovic et al., 2002). Third, simultaneous activation of several receptors may result in a crosstalk at the level of signaling events, potentially altering responses of some or all the receptors. In this regard, it was shown that membrane depolarization (as induced by activation of P2X receptors) can enhance P2Y-mediated Ca^2+^ responses, and that voltage-related potentiation of the P2Y1 receptor has an inverse relationship with agonist concentration (Gurung et al., 2008). Fourth, endogenously expressed receptors may be modulated by allosteric regulators or signaling pathways absent when the same receptor is expressed in heterologous systems (Coddou et al., 2011a). Finally, the possibility of a novel receptor subtype or splice variant cannot be discarded.

Notwithstanding the mechanism underlying this effect, our data suggest that the role of at least one of the P2 receptors active at 10^−7^–10^−4^ M [ATP] is to limit the responsiveness of the system over this range of ATP concentrations. Importantly, such behavior would result in a desensitization phase, and facilitate formation of a sharp threshold in response to ATP between 10^−4^ and 10^−3^ M.

### Study limitations

An important limitation of the current study is the application of P2 receptor characteristics obtained in studies of individual receptors in one cell type to the analysis of complex responses in another cell type. The following important assumptions were made during the initial stages of analysis. (1) We assumed that overexpression of an individual receptor in cells that do not normally express this receptor does not fundamentally change the characteristics of this receptor. While this may not be true, evidence of the contrary (that characteristics of P2 receptors change when overexpressed) has not yet been reported. Moreover, at this moment, there is no reliable way to obtain the concentration dependences of individual P2 receptors in an endogenous system due to overlapping action of the same ligand on different receptors. Therefore, we believe this assumption is important and unavoidable at the moment. (2) We assumed that characteristics of the receptors in related species of rodents (mice and rats) are similar and used them to model responses in a mouse cell line. While receptor characteristics may differ between mice and rats, limited data is available comparing responses. Of interest, we found that when receptors of rodent and human origin are compared, 4 out of 9 receptors have almost identical ATP concentration dependences, and 2 more have similar ATP concentration dependences (Figure 1). More studies are required to fully understand the differences between P2 receptors of different species.

## Conclusion

Taken together, our data suggest that, rather than having distinct individual roles, P2 receptors work in concert with both additive and inhibitory interactions. The ensemble yields a complex pattern of dependence on ATP concentration. This relationship is characterized by the presence of two thresholds: the first (at lower concentrations of ATP) likely relevant for tissue maintenance in response to moderate mechanical stimulation, and the second (at high concentrations of ATP) for anabolic responses to stronger mechanical stimulation and tissue damage. In mouse models, deficiency of individual P2 receptors studied to date have resulted in a bone phenotype; however, each of the reported phenotypes demonstrate unique characteristics, for example, reduced bone mass in P2Y1 and P2X7 receptor knockout mice, increased bone mass in P2Y2 and P2Y6 receptor knockout mice, and lower trabecular bone volume and increased cortical thickness in P2Y13 receptor knockout mice (Wang et al., 2013). In future studies, it will be important to examine how dependence on ATP concentration is affected by the absence of individual P2 receptors. Moreover, it would be interesting to test for heterologous desensitization among P2 receptors, as predicted by our model. Such information may allow the design of targeted experiments aimed at determining how change in overall [ATP] dependence affects bone adaptation to low, intermediate or high mechanical strains.

## Author contributions

Study conception and design: SX, PG, SD, SK. Acquisition of data: SX, MG. Analysis and interpretation of data: SX, MG, SD, SK. Drafting of manuscript: SX, SK. All authors contributed to the critical revision of manuscript and approved the final version to be published.

## Funding

This research was funded by the Canadian Institutes of Health Research (CIHR) grants number 77643 and 102542, and by the Natural Sciences and Engineering Research Council of Canada grant number RGPIN-2015-05579.

### Conflict of interest statement

The authors declare that the research was conducted in the absence of any commercial or financial relationships that could be construed as a potential conflict of interest.
