# Supplementary material for: Modeling Interactions among Individual P2 Receptors to Explain Complex Response Patterns over a Wide Range of ATP Concentrations
Source: Front Physiol. 2016 Jul 13;7:294. doi: 10.3389/fphys.2016.00294 (PMC4942464; doi:10.3389/fphys.2016.00294)
Supplement: Supplementary file 1 [file DataSheet1.pdf]

Supplementary information for the manuscript by S Xing, MW Grol, P Grutter, SJ Dixon, SV Komarova, (2016) *Modeling Interactions among Individual P2 Receptors to Explain Complex Response Patterns over a Wide Range of ATP Concentrations.* Frontiers in Physiology

**Supplementary Matlab Code 1**

Amplitude, Duration and Amount analysis of the experimental data of individual osteoblasts

```
close all
clear all
clc
cd E:\Masters\Masters\ATPexpdata\Single Cell Trace Amplitude Analysis
atpdata= xlsread('ATP Single Cell Traces 08 Apr 2011','ATP (10
mM)','6:1205'); % opens excel files reading only numerical values\
numcol=size(atpdata,2); % number of columns
time= atpdata(:,1); % take first column data
final=[];
%% Data analysis starts here%%
for i=2:2:(numcol-1); % select the not normalized data
nintensity =atpdata(:,i);
figure()
plot(time,atpdata(:,i));
title(i)
%% to get the basal values%%
basalvalues = atpdata(195:295,i);
avgbasal=mean(basalvalues);
stdbasal=std(basalvalues);
%% to get the amplitude %%
[amplitudex,amplitudey]=ginput(2); % select the range with a cross for which
amplitude will be calculated
ampxone= amplitudex(1,1);
rampone= find(time> ampxone-0.75 & time< ampxone+0.75);% to find which row is
the picked value in excel file
ampxtwo= amplitudex(2,1);
ramptwo= find(time> ampxtwo-0.75 & time< ampxtwo+0.75);
amprange=atpdata(rampone:ramptwo,i);
amp{i/2}= amprange; % {}marks the index; (i)/2 makes the array without all
the empty columns
maximumvalue=max(amprange);
amplitude = maximumvalue-avgbasal;
%% test if it is a real response%%
if amplitude > stdbasal*4
 disp('There is response')
 normatp=atpdata(:,i)./avgbasal; % normalize the data to 1
 normatpdata{i/2}= normatp;
 figure()
 plot(time,normatp)
 title 'normal'
 finalatp=normatp - mean(normatp(195:295)); %to offset the basal from 1 to 0
 finalatpdata{i/2}=finalatp;
 figure()
 plot(time, finalatp)
 title 'final'
 %% calculate final basal%%
 fbasrange=finalatp(195:295);
 finalbasal{i/2}=fbasrange;
```

```
 meanbas= mean(fbasrange);
 %% calculate amplitude%%
 [finalampx,finalampy]=ginput(2);
 finalampxone= finalampx(1,1);
 rfinalampone= find(time> finalampxone-0.75 & time< finalampxone+0.75)
 finalampxtwo= finalampx(2,1);
 rfinalamptwo= find(time> finalampxtwo-0.75 & time< finalampxtwo+0.75)
 finalamprange=finalatp(rfinalampone:rfinalamptwo);
 finalamp{i/2}= finalamprange;
 finalmaxvalue=max(finalamprange);
 finalamplitude=finalmaxvalue-meanbas;
 %% calculate duration%%

%find the duration of the curve: taking the half amplitude and find the
coordinates associated with that value
 f=fit(time(rfinalampone:rfinalamptwo),finalamprange,'gauss4');
 [ci,gofi]=fit(time(rfinalampone:rfinalamptwo),finalamprange,'gauss4');
 figure()
 plot(f,time(rfinalampone:rfinalamptwo),finalamprange);
 t=time(rfinalampone:rfinalamptwo);

 %from fit

 [maximoF,index1F]=max(ci(t))
 halfmaxF=maximoF/2;
 myXLF=t(1:index1F);
 myYLF=ci(myXLF);

 myIndicesLF=find(myYLF<=halfmaxF);
 myX1LF=myXLF(myIndicesLF);
 tLF=myX1LF(end);

 myXRF=t(index1F:end);
 myYRF=ci(myXRF);

 myIndicesRF=find(myYRF>=halfmaxF);
 myX1RF=myXRF(myIndicesRF);
 tRF=myX1RF(end);
 deltaF{i/2}=tRF-tLF;
 durationF=tRF-tLF;
    title(i)

 %% calculate amount%%
 amountrange = finalatp(rfinalampone:rfinalamptwo);
 finalampyone=finalampy(1,1);
 finalamount= amountrange-finalampyone;
 figure()
 plot(time(rfinalampone:rfinalamptwo),finalamount)
 finalamt{i/2}= finalamount;
 amount= trapz(time(rfinalampone:rfinalamptwo),finalamount);
else
 disp('There is no response')
end
%% Put all the information into a matrix called "final"
final((i)/2,1)= avgbasal;
final((i)/2,2)= stdbasal*4;
```

```
final((i)/2,3)= amplitude;
final((i)/2,4)=meanbas;
final((i)/2,5)=finalamplitude;
final((i)/2,6)=amount;
final((i)/2,7)=duration;
final((i)/2,8)=durationF;
end
```

**Supplementary Matlab Code 2**

Obtaining the Hill equation of individual P2 receptors

```
close all
clear all
clc
cd /Users/Stella/Desktop/ReceptorRawdata
%% P1X1
receptordata= xlsread('P2Raw','P2X1','2:8'); % opens excel files reading only
numerical values\
numcol=size(receptordata,2); % number of columns
atpconc=receptordata(:,1);% take first column data
response= receptordata(:,2); % take the second column data
errorbars= receptordata(:,5);

s = fitoptions('Method','NonlinearLeastSquares',...
 'Lower',[0,0],...
 'Upper',[Inf,1],...
 'Startpoint',[0.001,1e-6]);
f = fittype('(x^n)/((u)^n+x^n)','options',s);
[ci,gofi] = fit(atpconc,response,f);
ci
gofi

figure()
semilogx(atpconc,response,'k.','MarkerSize',15);
box off
hold on
errorbar(atpconc,response,errorbars,'k.','MarkerSize',15);
x1=[1E-9:1E-8:1E-2];
plot(x1,ci(x1),'color',[1 0.8 0],'LineWidth',2);
xlim([1E-9 0.01]);
ylim([0 1.2]);
xlabel('ATP Concentration (M)','FontSize',16,...
 %'FontName','Arial')
ylabel('Response(% maximum response)','FontSize',16,...
 %'FontName','Arial')
title('P2X_1','FontSize',16,...
 'FontName','Arial')
```

**Supplementary Matlab Code 3**

Fitting the linear combination of P2 receptor functions to the [ATP] dependent peak amplitude of calcium response

```
clear all
```

```
%% Use the single cell data I got from London, I analyzed to get the amount:
area under the curve %%
%% raw data
atpconc=[0.000000001;0.00000001;0.0000001;0.000001;0.00001;0.0001;0.0002;0.00
03;0.0005;0.001];
avgamplitude=
[1.61587153100000,2.22973987100000,3.40114272300000,2.66473611400000,1.856800
61100000,1.21627212800000,1.06142041200000,2.28626008400000,3.59074928600000,
2.93142880400000];
E=
[0.877857375,1.36611442200000,1.00259447200000,1.27999448600000,0.62503551200
0000,0.722449080000000,0.420549322000000,0.730396613000000,0.591134405000000,
0.890312201000000];
%% Nomalized data
concfinal=atpconc;
ampfinal=(avgamplitude'./3.590749286); %this is to normalize the exp data to
1 so it matches with the receptors data
sdfinal=E(1:4)./3.590749286;
%% there are 3 p2X receptors are 3 p2Y receptors present on osteoblasts:
P2X2,5,7 and P2Y1,2,4
% p2y2=(x.^1.371)./((2.862e-06^1.371)+x.^1.371);
% p2x5=(x.^1.311)./(((7.801e-06)^1.311)+x.^1.311);
% p2y1=(x.^0.4551)./((2.682e-07)^0.4551+x.^0.4551);
% p2x2=(x.^1.305)./(((8.244e-06)^1.305)+x.^1.305);
% p2y4=(x.^0.995)./(((1.202e-06)^0.995)+x.^0.995);
% p2x7=(x.^2.183)./(((0.0001309)^2.183)+x.^2.183);
%% fit the p2xy receptor to the amount %%

s = fitoptions('Method','NonlinearLeastSquares',...
 'Lower',[-inf,-inf],...
 'Upper',[inf,inf],...
 'Startpoint',[1,1]);
f = fittype('ky1*(x.^0.4551)./((2.682e-07)^0.4551+x.^0.4551)+
kx7*(x.^2.183)./(((0.0001309)^2.183)+x.^2.183) ','options',s);
[ci,gofi] = fit(concfinal,ampfinal,f);
ci
gofi
coeff=coeffvalues(ci);
figure()
data=semilogx(concfinal,ampfinal,'k.','MarkerSize',15);
hold on
errorbar(concfinal,ampfinal,sdfinal,'k.',...
 'MarkerSize',15,...
 'Linewidth',2);
x1=[1E-9:1E-9:1E-3];
fit=plot(x1,ci(x1));
colours=colormap(hsv);
set(fit,'Color',colours(1,:),'LineWidth',2);
xlabel('ATP concentration [M]','FontSize',16,...
 'FontName','Arial');
ylabel('Normalized Amplitude of calcium released','FontSize',16,...
 'FontName','Arial');
```
